# Analysis of Active Components and Transcriptome of *Freesia refracta* Callus Extract and Its Effects against Oxidative Stress and Wrinkles in Skin

**DOI:** 10.3390/ijms25158150

**Published:** 2024-07-26

**Authors:** Euihyun Kim, Morgane De Tollenaere, Benedicte Sennelier, Carole Lambert, Anais Durduret, Soo-Yun Kim, Hyo-Hyun Seo, Jung-Hun Lee, Amandine Scandolera, Romain Reynaud, Sang-Hyun Moh

**Affiliations:** 1 Plant Cell Research Institute of BIO-FD&C Co., Ltd., Incheon 21990, Republic of Korea; hyun9214@snu.ac.kr (E.K.); sykim@biofdnc.com (S.-Y.K.); hhseo@biofdnc.com (H.-H.S.); jhlee@biofdnc.com (J.-H.L.); 2 Givaudan France SAS, Route de Bazancourt, 51110 Pomacle, France; morgane.de_tollenaere@givaudan.com (M.D.T.); anais.durduret@givaudan.com (A.D.); amandine.scandolera@givaudan.com (A.S.); 3 Givaudan France Naturals, 250 rue Pierre Bayle, BP 81218, 84911 Avignon, France; benedicte.sennelier-portet@givaudan.com; 4 Givaudan France SAS, Bâtiment Canal Biotech 1, 3, Rue des Satellites, 31400 Toulouse, Franceromain.reynaud@givaudan.com (R.R.)

**Keywords:** *Freesia refracta* callus extract, antioxidant, anti-aging, cosmetic ingredient, plant cell culture

## Abstract

*Freesia refracta* (FR), a perennial flower of the Iris family (Iridaceae), is widely used in cosmetics despite limited scientific evidence of its skin benefits and chemical composition, particularly of FR callus extract (FCE). This study identified biologically active compounds in FCE and assessed their skin benefits, focusing on anti-aging. FR calli were cultured, extracted with water at 40 °C, and analyzed using Centrifugal Partition Chromatography (CPC), Nuclear Magnetic Resonance (NMR), and HCA, revealing key compounds, namely nicotinamide and pyroglutamic acid. FCE significantly increased collagen I production by 52% in normal and aged fibroblasts and enhanced fibroblast–collagen interaction by 37%. An in vivo study of 43 female volunteers demonstrated an 11.1% reduction in skin roughness and a 2.3-fold increase in collagen density after 28 days of cream application containing 3% FCE. Additionally, the preservation tests of cosmetics containing FCE confirmed their stability over 12 weeks. These results suggest that FCE offers substantial anti-aging benefits by enhancing collagen production and fibroblast–collagen interactions. These findings highlighted the potential of FCE in cosmetic applications, providing significant improvements in skin smoothness and overall appearance. This study fills a gap in the scientific literature regarding the skin benefits and chemical composition of FR callus extract, supporting its use in the development of effective cosmeceuticals.

## 1. Introduction

*Freesia refracta (Jacq.) Klatt* (FR), a perennial flower belonging to the Iridaceae family, is commonly cultivated as a decorative plant in South Africa. Typically occurring in vivid yellow, varieties in white, red, purple, and pink varieties also exist. FR is considered as a “silent flower,” meaning that it is not possible to obtain any absolute or essential oil. Nevertheless, its perfume is famous in the fragrance industry and can be reproduced synthetically. In fact, the artificial aromatic essence of FR is used extensively in cosmetics [1,2,3]. However, scientific evidence supporting the skin benefits of FR remains limited, with no scientific publications having described the chemical composition of FR callus extracts (FCE). Furthermore, there have been few reports on its effects in the fields of medicine and pharmacology. By contrast, terpineol and its isomers, which are commonly found in flowers [4] and herbs, such as FR, narcissus oregano, and rosemary, have been reported to have a wide range of pharmaceutical and biological properties [5,6,7]. In a previous study, Zhu et al. described the composition of flavonoids and anthocyanins in the petals of Freesia [8]. Most of these bioactive compounds are sourced directly from plants, and instances of extraction from plant-derived calli for application in cosmetics or pharmaceuticals are particularly scarce.

Callus culture technology plays a crucial role in achieving standardized mass production efficiency without harming plants [9,10,11], and involves the proliferation of undifferentiated cells from wounded explant tissues isolated from various plant organs [12]. Building on these points, for several decades, research on plant calli has focused on genetic editing, regeneration, or plasmolysis. However, research on extraction from plant calli and subsequent evaluations thereof remains scarce, with even fewer studies exploring this avenue, due to the limited diversity in plant species. Some studies have demonstrated the effects of plant callus extracts both in vitro and in cosmetics. Tadhani et al. indicated that calli contain more phenols and flavonoids than leaves [13]. Santos et al. demonstrated that callus extracts have analgesic effects in rodent species [14]. Moreover, the callus provides efficient protection in vitro, including against oxidative stress, and enhances the viability of human keratinocytes [15]. We recently reported on the effect of a lotus-derived callus extract on human skin, which has been shown to be critical for skin whitening [16]. Likewise, extracts from plant calli have been proven to have significant effects in a wide range of studies, which implies that FCE may also have similar effects. This suggests that extracts of plant calli may have more byproducts than the extracts from leaves or plantlets.

To achieve a high range of phytochemical profiles with use of the most natural solvent (water), water extraction was performed at 40 °C for 4 h on freesia callus plant dried cells. Water is considered as the greenest solvent (no toxicity or inflammability, environmentally friendly, and freely available), and a temperature in a range of 40–50 °C avoids altering bioactive phytochemicals [17]. Moreover, the water extract was well tolerated for better solubilization in culture media for the in vitro evaluation of skin cell activity. Additionally, when producing extracts, not only the solvent used but also the temperature at which the extraction is conducted is an important consideration. Although extraction has been performed at 121 °C in some cases, temperatures below 60 °C are necessary to prevent the destruction of the molecule of interest due to excessively high temperatures. For example, Mokhtari et al. performed casein hydrolysis within a temperature range of 40–60 °C for 4–6 h in order to isolate peptides [18]. Furthermore, Salehan et al. performed the extraction of gallic acid from *Labisia pumila* at 50 °C for 6 h. Although this condition was not optimal, the authors achieved enhanced yields. The potential influence of the best extraction performance may be due to the decomposition of the target compound [19]. Based on the previously reported effects of FR, in this study, we aimed to identify the biologically active compounds and skin benefits of FCE, and hypothesized that this extract would show enhanced effects, particularly in wrinkle improvement, skin antioxidation, and anti-aging.

## 2. Results

### 2.1. Chemical Profiling and Quantification of Main Actives Molecules: Nicotinamide and Pyroglutamic Acid

**Figure 1:**
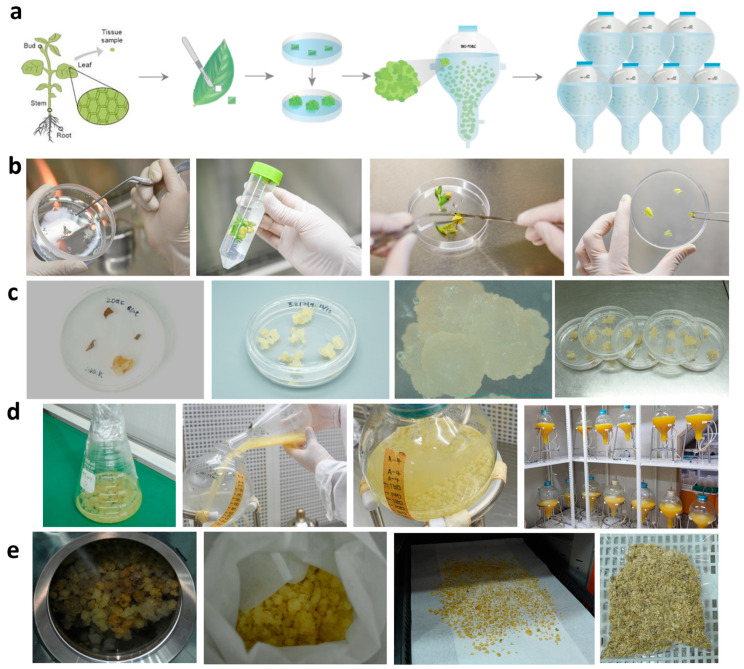
(**a**) A graphical procedure of FCE production. (**b**) The FR was firstly germinated in a dish filled with germination medium, then a leaf of the FR plantlet was sliced into small pieces. The leaf slices tissues were plated on callus induction medium. (**c**) When the first calli were observed from the tissues, they were then transferred to a callus proliferation medium. (**d**) The culture of proliferated callus was scaled up to liquid culture with agitation, then up to 10 L bioreactors. (**e**) Subsequently, the callus was harvested by dehydration and heat dryer.

**Figure 2:**
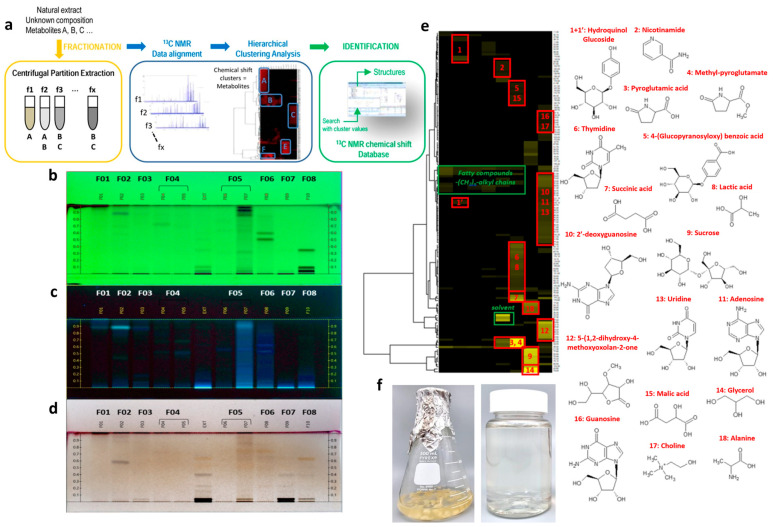
(**a**) A series of processes of chemical profiling methodology was applied to characterize compounds in FCE. Then, thin-layer chromatography (TLC) profiles was obtained for the identification process; (**b**) 254 nm, (**c**) 366 nm, and (**d**) visualized chromatography after vanillin/H_2_SO_4_ reagent spraying and heating. (**e**) 13C NMR chemical shift clusters (yellow color) obtained by applying HCA on CPC fractions of FCE and the chemical structures of major compounds identified in FCE. (**f**) cultured Freesia callus and its extract. NMR: nuclear magnetic resonance; TLC: thin-layer chromatography profiles; FCE: Freesia callus extract; HCA: hierarchical clustering analysis.

The prepared FR was sterilized and used for callus induction. The induced FR calli were used for suspension cell cultivation in a liquid medium. The FR cells were then mass-cultivated in bioreactors. Subsequently, the cells were heat-dried and extracted (Figure 1b–e and Figure 2f). The major compounds of FCE were identified using a dereplication method that combines CPC, NMR analyses, and HCA (Figure 2a) [20]. The CPC fractionation of FCE resulted in the production of eight fractions, and the major metabolites was subsequently identified (Figure 2b–d and Table 1) of the simplified chemical composition in increasing order of polarity and without any loss of material. All fractions analyzed by NMR and ^13^C NMR spectra were submitted to Hierarchical Clustering Analysis for the classification and visualization of ^13^C NMR metabolite fingerprints. As illustrated in Figure 2e, the major chemical shift clusters shown in yellow on the heat map corresponded to the major metabolites of FCE. Using an in-house database containing the predicted chemical shift values of natural metabolites, the correlated chemical shifts of the most intense clusters (3 and 4) were assigned to pyroglutamic acid (3) and methyl-pyroglutamate (4). The identification of these molecules was confirmed by verifying all 1H-13C and ^1^H-^1^H correlations in the HSQC, HMBC, and COSY spectra of F5, F6, and F8. Using the same method, the correlated chemical shifts of Cluster 2 were assigned to the nicotinamide detected in F4. Moreover, along with the result of BPI (ESI-) chromatogram , the elution fractions (representing 3.6% of the injected mass) were found to be mainly composed of a mixture of fatty acids, fatty alcohols, hydroquinol–glucopyranoside (arbutin), and nicotinamide, while the two last fractions that were recovered by the extrusion step (representing 96.3% of the injected mass) were mainly composed of sucrose, glycerol, nucleosides, and organic acids among the categorized molecules. In particular, the identification of nicotinamide, pyroglutamic acid, and methyl-pyroglutamate was notable (Table 1 and Table 2).

### 2.2. Quantification of Nicotinamide and Pyroglutamic Acid by Liquid Chromatography Using Mass Detection or UV Detection

Based on previous findings relating to the use of these molecules for cosmetic applications, quantitative analyses were performed to determine the content of pyroglutamic acid (324 mg/kg) and nicotinamide (163 µg/kg). However, the methyl-pyroglutamate content could not be quantified. These results indicated that these substances may exert an influence in subsequent experiments.

### 2.3. Boost of Collagen I Production

**Figure 3:**
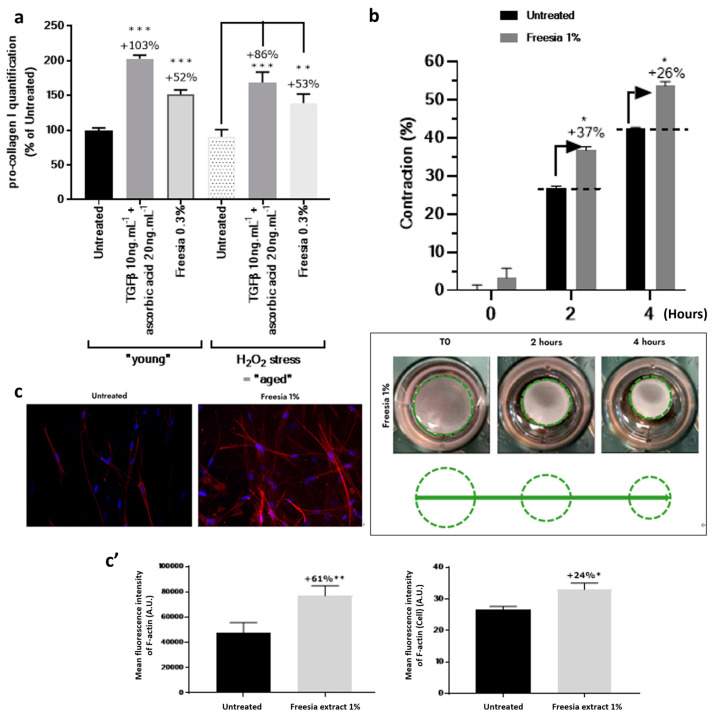
Evaluations of FCE at the in vitro level. The effect of FCE was assessed by (**a**) type I pro-collagen quantification using ELISA, (**b**) collagen contraction assay using matrices with 1% FCE, and (**c**–**c’**) immunostaining of F-actin with a comparison of intensities between the control and 1% FCE-treated group. The experiments were performed in at least three replications. Bars with asterisks indicate significant differences (* *p* < 0.05, ** *p* < 0.01, *** *p* < 0.001). FCE: Freesia callus extract.

Next, we evaluated the effect of FCE on collagen I production using an in vitro model to study its anti-aging activity. An in vitro model based on human primary fibroblasts exposed to 0.3% FCE under basal conditions or after premature aging induction by H_2_O_2_ treatment was used. Under basal conditions, the positive control (TGF-β + ascorbic acid) significantly boosted the collagen I production by +103% (*p* < 0.0001), validating our model. Under similar conditions, 0.3% FCE significantly increased the collagen I production by +52% (*p* < 0.0001). Interestingly, when exposed to age-mimicking conditions, the presence of FCE at a concentration of 0.3% demonstrated an equivalent performance, resulting in the significant restoration of collagen I production with a remarkable increase of +53% (*p* < 0.001, Figure 3a). In conclusion, our results provide clear evidence of the strong and significant anti-aging activity of FCE, as it effectively enhanced collagen I neosynthesis under both normal and aged conditions.

### 2.4. Reinforcement of the Interactions between Fibroblasts and Collagen Fiber

Continuing our investigation into the interaction between fibroblasts and collagen fibers and their role in skin structure and surface improvement, we used lattice collagen treated with 1% FCE and collagen contraction via diameter measurements as an indirect strategy to evaluate the interactions and performance of fibroblasts with collagen. We observed progressive and significantly better collagen contraction with 1%. In comparison, the 1% FCE-treated samples showed a +37% and +26% contraction after 2 and 4 h, respectively (*p* < 0.05, Figure 3b). The corresponding images demonstrate the strong ability of FCE to activate the interaction between fibroblasts and collagen fibers. Under the same conditions, we analyzed actin expression to visualize and analyze the tensile force expressed by fibroblasts during interactions with collagen fibers to confirm previous observations. Via the specific immunodetection of F-actin, 0.1% FCE was found to significantly increase the intensity of F-actin expression in fibroblasts, showing a +61% and +24% increase for global F-actin expression analysis and F-actin expression by cell analysis, respectively (*p* < 0.001 and *p* < 0.05, respectively; Figure 3c,c’). These results demonstrate that FCE improved fibroblast–collagen interactions, promoting better dermal contraction, as indicated by the improved F-actin network and expression for soothing skin properties.

### 2.5. Delivery of Smoothness on the Skin Surface after 28 Days (In Vivo)

**Figure 4:**
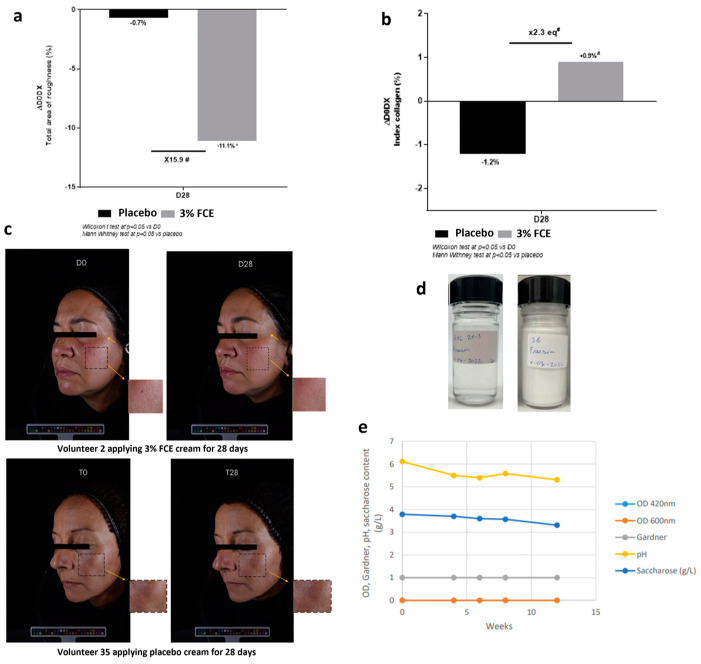
Clinical assessment of the FCE. A total 43 women participated in the clinical tests and the placebo, in which the FCE is absent, was used as the control group in all tests. Several clinical tests were performed for (**a**) skin roughness, (**b**) collagen test, and (**c**) improvement in wrinkles when 3% FCE was applied. Moreover, as shown in (**d**,**e**) the preservability of the temporary products used in the clinical tests was evaluated through tests on OD values, Gardner, pH, and sucrose (g/L). Bars with asterisks indicate significant differences (* *p* < 0.05). FCE: Freesia callus extract.

A significant improvement was observed in the properties of the skin surface among volunteers who applied a cream containing 3% FCE, as evidenced by a reduction in skin roughness of 11.1% compared with the baseline measurement (D0) 28 days after application (*p* < 0.05, Figure 4a). Moreover, the results demonstrated a considerably greater smoothing effect on the skin surface with the use of 3% FCE compared to the placebo. Specifically, we observed a 15.9-fold reduction in skin roughness when comparing 3% FCE to the placebo. In addition, we analyzed the collagen index to further validate the anti-aging effects of FCE at the clinical level. The results revealed a significant increase in the collagen index after 28 days of the twice-daily application of a cream containing 3% FCE compared with a placebo. This resulted in a 2.3-fold increase in collagen density, indicating a substantial improvement in the anti-aging properties of the skin (Figure 4b). Furthermore, to visually demonstrate the enhanced skin smoothing effect, we utilized illustrative pictures obtained using the ColorFace^®^ method. In Figure 4c, the skins of the representative volunteers (volunteers 2 and 35) show marked differences in roughness, consistent with Figure 4a,b.

Next, additional preservation tests on these cosmetics were performed. To this end, we prepared toners and creams with 3% FCE and monitored their preservation status for approximately 12 weeks (Figure 4d). No changes were observed in the O.D values or Gardner’s changes, and only minimal changes in sucrose and pH were found (Figure 4e). These results indicate that there are no issues in developing cosmetics using 3% FCE.

### 2.6. Transcriptome Analysis of FCE

**Figure 5:**
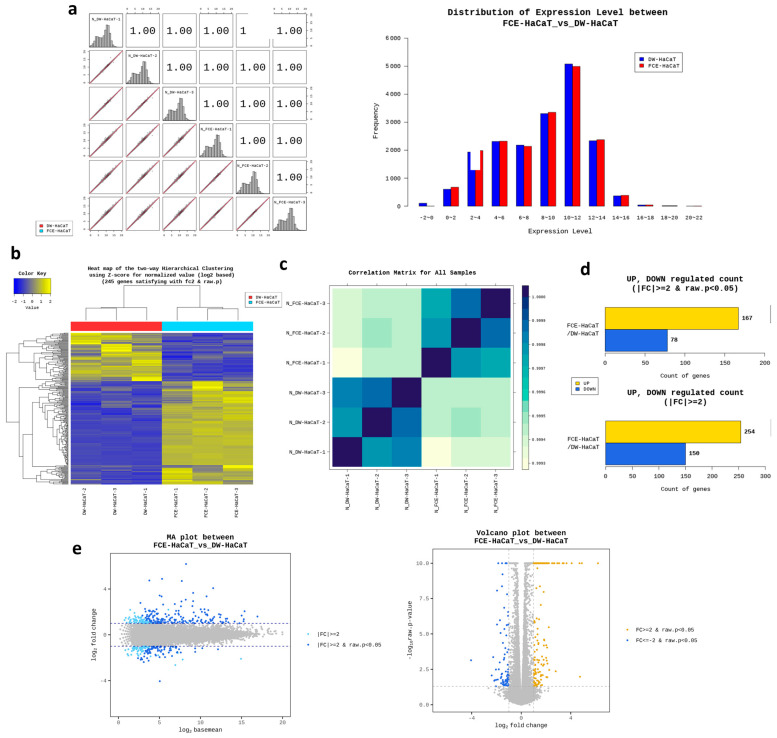
The results of transcriptome analysis on effects of FCE on human keratinocyte. Several categories of analysis such as (**a**) the distribution of the gene expression level; (**b**) the heat map of the two-way hierarchical clustering using Z-score for normalized value; (**c**) correlation matrix; (**d**) distribution of up/down-regulated DEGs; and (**e**) volcano plots of the DEGs were applied. FC: Fold change; DW: Distilled water (control); FCE: Freesia callus extract.

**Figure 6:**
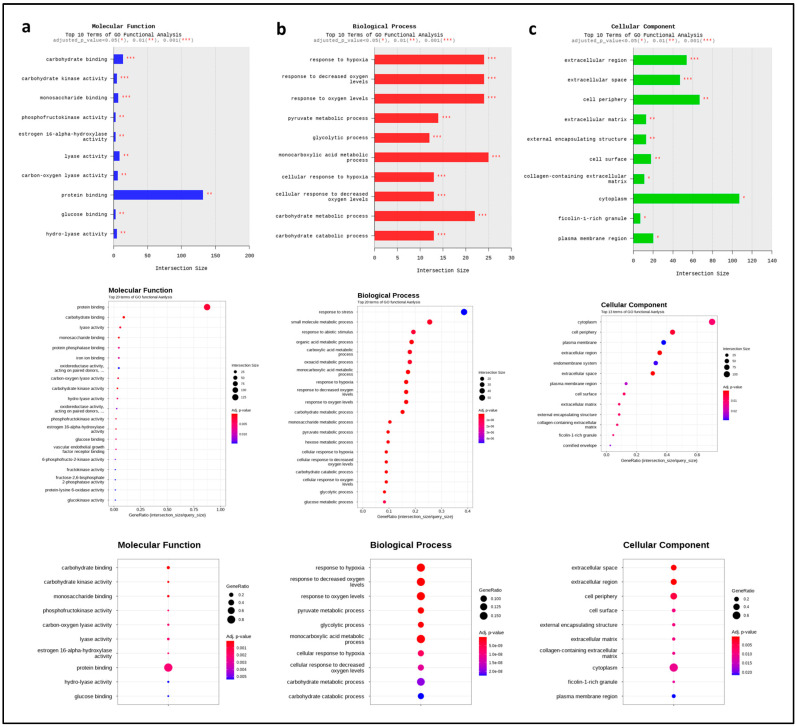
Result of gene ontology (GO) of effects of FCE on human keratinocyte. The GO term was categorized as (**a**) molecular function, (**b**) biological process, and (**c**) cellular component. Differences among the terms were shown as dots with intersection sizes, then the *p*-value was adjusted and demonstrated with color. The graph shows only top 10 or 20 GO functional analysis. GO: Gene ontology.

Lastly, we conducted transcriptome analysis by treating with 1% FCE. We compared the gene expression between the group treated with 1% FCE and the control group treated with DW, the solvent of FCE. First, after obtaining the raw data, we made the data comparable through a series of data trimming and normalization, logarithm, and distribution by zero count numbers (Figures S1, S2, and Figure 5a). Subsequently, the expression patterns of differentially expressed genes (DEGs) were confirmed through a heatmap, which showed clearly different and opposing gene expression patterns between the FCE-treated group and the control group (Figure 5b). This experiment was conducted with three biological replications, and the similarity of each sample used in the experiment was shown through a correlation matrix (Figure 5c). Additionally, the amount of upregulation and downregulation of the total DEGs was represented using a volcano plot, as shown in Figure 5d,e. As a result, a total of 254 genes were upregulated and 150 genes were downregulated. Among these, considering a significance condition with a *p*-value of less than 0.05, 167 genes were upregulated and 78 genes were downregulated. Through these DEGs, we analyzed Gene Ontology (GO) terms. The results showed that, in terms of molecular function, protein binding had the most significant change (Figure 6a), and in biological processes, the response to hypoxia, response to oxygen levels, and carbohydrate metabolic process had the most significant changes (Figure 6b). Lastly, in the case of cellular components, the most significant changes in gene expressions were related to the cytoplasm, cell periphery, and extracellular region (Figure 6c). For example, our analysis indicated that genes such as BNIP3 (BCL2 interacting protein 3), STC1 (Stanniocalcin 1), ANGPTL4 (Angiopoietin-like 4), FLG (Filaggrin), MIR210 (MicroRNA 210), CYP1A1 (Cytochrome P450 family 1 subfamily A member 1), EDN2 (Endothelin 2), HK2 (Hexokinase 2), and SLC2A3 (Solute carrier family 2 member 3) showed significant fold changes. They are known to function in antioxidant, whitening, and wrinkle-related processes. Therefore, these results indicate that the FCE treatment most significantly affects the gene expression related to oxidative stress within the cell, and this may show similar tendencies in our clinical tests and in vitro tests.

## 3. Discussion

Several studies have focused on regeneration and protoplast induction in FR callus [21,22,23]. However, despite considerable research related to extracts or flavonoids derived from FR [8,24] and their associated metabolites, research on the flavonoids or metabolites present in extracts derived from FR calli is currently lacking. The plant callus is typically a mass of unorganized plant parenchyma cells that form around plant wounds. Recently, studies demonstrated that plant callus extracts affect human cells. Burannasudja et al. demonstrated the antioxidant and anti-aging properties of callus extracts from *Centella asiatica* in human foreskin fibroblasts [25], while Kwon et al. found that callus-derived shikimic acid could convert human dermal fibroblasts into multipotent skin-derived precursor cells [26]. Based on these findings, we hypothesized that extracts from FR calli may also have beneficial effects on human cells in vitro and skin in vivo and evaluated the effective metabolites from its extract.

By controlling the nutrients, plant growth regulators, inducers, and culture conditions, callus culture can be optimized uniformly [27,28]. Cell suspension culture enables the relatively rapid harvesting of biomass within 4–8 weeks [28]. Cultivating calli in a bioreactor allows for the homogenized and controlled growth of plant cells. Plant cell fractions extracted from cultured cells contain essential components that can promote health [29,30]. Consequently, in vitro cultured organs can yield substances with higher activities than those obtained from plants grown under natural conditions. In particular, when manufacturing antioxidant, anti-wrinkle, or functional cosmetics, plant-derived extracts are commonly added. However, upon closer examination, it becomes evident that there are numerous variables to consider regarding the addition of extracts, such as the precise concentration, batch of cultivated plants, and the culture environment. Nonetheless, plant calli present an advantage in ensuring consistency in both experimental and product batches, as they are produced under the same medium composition, within the same timeframe, and in controlled environments.

Through transcriptome analysis, we could infer the effects of FCE. From Figure 5 and Figure 6, it was observed that FCE mainly showed significant gene changes related to response to hypoxia, oxidative stress, and carbohydrate metabolic processes, which occur in the extracellular region and cytoplasm, and that protein binding had the highest rate of gene changes. For example, Angiopoietin-like 4, Cytochrome P450 family 1 subfamily A member 1, Solute carrier family 2 member 3, and Endothelin 2 are known to have functions related to skin whitening or wound healing effects, and showed significantly high fold changes in our experiments [31,32,33]. Similarly, BCL2 interacting protein 3, MicroRNA 210, Hexokinase 2, and Filaggrin also exhibited very high fold changes and are associated with antioxidant, wrinkle reduction, inflammation, and wound healing effects [34,35,36]. Interestingly, to briefly summarize these results, it may be interpreted that a signaling pathway or some factors were related to the oxidative stress and it occurred within the cytoplasm and outside of the cells. One possible explanation for this is the Nuclear factor erythroid-2-related factor 2 (Nrf2) signaling pathway. The factor Nrf2 is normally kept in the cytoplasm, and upon oxidative stress or external stimuli, it translocates into the nucleus, producing various antioxidant-related elements (AREs) such as catalase, heme oxygenase 1, glutathione peroxidase 1, NAD(P)H quinone oxidoreductase 1 (NQO1), and superoxide dismutase (SOD) [37,38,39]. These elements reduce the oxidative stress occurring in cells. Furthermore, the Nrf2 signaling pathway is known to be effective in wound healing, inflammation, and wrinkle improvement [40,41]. Therefore, the fact that we could observe such effects through transcriptome analysis suggests that not only morphological effects but also important mechanisms like Nrf2 at the molecular level might be involved. Indeed, among the related genes, those upregulated include ARE elements such as NAD(P)H quinone oxidoreductase 1, SOD1, SOD2, anti-aging-related genes, such as lamin A/C and insulin-like growth factor 1 receptor, were also regulated, and genes related to wound healing like filaggrin, vascular endothelial growth factor A, and tissue inhibitors of metalloproteinases 1 were significantly upregulated. Additionally, in accordance with our results, it was confirmed that FCE regulates many genes related to the antioxidant effects and the effects we aimed to observe.

Skin aging can be classified into various categories, including photoaging, chronological aging, galactose-induced aging, and inflammation [42,43,44]. The aging of the skin can manifest as an uneven texture, wrinkles, and loss of elasticity, all of which are considered clinical indicators of aging [45]. Given the aforementioned effects of callus extracts, we applied FCE to human skin. Our results, obtained through in vitro and clinical tests following the guidelines for stability testing of cosmetic products suggested by the Ministry of Food and Drug Safety, demonstrate the significant anti-aging effectiveness of FCE on human skin. To obtain FCE, FR calli were first induced under sterile conditions (Figure 1) before cultivating on a large scale and treating the cells with the extract. The results presented in Figure 2, Table 1, Table 2, and Table S1 can be explained by a previous study in which Chen et al. extracted and detected several flavonoids using High-Performance Liquid Chromatography (HPLC) from *Dysosma versipellis*, suggesting that the callus itself may produce useful flavonoids [46]. Moreover, FR has been reported to contain flavonol derivatives, such as quercetin and kaempferol, identified using HPLC-mass spectrometry [47]. In the field of cosmeceutical research, it is widely known that quercetin and kaempferol exhibit antioxidant, collagen synthesis, and moisturization activities [48,49]. Herein, although the substances we identified were not flavonoids, compounds with similar roles existed, including antioxidant and anti-inflammatory properties. For example, nicotinamide, a form of vitamin B3, is known for its anti-inflammatory [50], skin promoting [51], and anti-aging properties [52]. Additionally, pyroglutamic acid, derived from glutamic acid, is known for its antioxidant properties [53].

Therefore, the positive effects of FCE could be attributed to the compounds (nicotinamide, pyroglutamic acid, and methyl-pyroglutamate) that we previously identified by NMR. To the best of our knowledge, this study is the first to report on the detection of nicotinamide, pyroglutamic acid, and methyl-pyroglutamate in extracts from *Freesia* plants, including calli. Thus, there is a possibility that our discovery of these compounds in *Freesia* callus extract (FCE) may be a novel and the first finding of its kind.

Among these two compounds, nicotinamide has been studied extensively for over two decades. As a result, the effects of nicotinamide on the skin are well known. For example, it has been reported that nicotinamide acts in the process where aged fibroblasts secrete less collagen compared to younger cells [54]. Similarly to our research, Bissett et al. applied nicotinamide as a topical treatment in a clinical study and demonstrated its anti-aging and anti-wrinkle effects on human facial skin [55]. Additionally, studies have also previously reported on its effects on pigmentation, redness, and the skin barrier [51,56,57]. By contrast, pyroglutamic acid has been reported to exhibit antibacterial [58], antifungal [59], and antithrombotic activities [60]. Unfortunately, information on pyroglutamic acid is limited; however, based on the results showing the effectiveness of FCE, we anticipate that this substance could also be beneficial beyond the aforementioned effects. Further molecular analyses will be needed to better understand the efficacy of FCE. Nonetheless, the in vitro and clinical test results obtained in this study demonstrate the potential of FCE to restore and provide anti-aging benefits to human skin. Although numerous plant-based raw materials are used in cosmetics, this study proposes a novel methodology that utilizes plant calli and their extracts as a promising source of cosmetic materials.

## 4. Conclusions

In this study, FCE demonstrated anti-aging effects by significantly increasing collagen I production in both young and prematurely aged fibroblasts. Additionally, this active ingredient was also found to enhance the interaction between fibroblasts and collagen fibers, leading to improved collagen fiber contraction. This improvement was further supported by the overexpression of F-actin in the fibroblasts.

To validate the in vitro findings and establish a clinical correlation, we clinically evaluated 43 female volunteers. The results confirmed that the application of 3% FCE significantly improved the overall appearance of the skin, particularly in terms of a noticeable smoothing effect. This improvement was closely associated with increased collagen production, providing a unique and optimal anti-aging benefit. These findings strongly suggest that FCE possesses remarkable efficacy in restoring human skin and has potential applications in the field of cosmeceuticals.

## 5. Materials and Methods

### 5.1. Chemicals, Reagents, and Ethical Statement

The inventory of chemicals and reagents utilized throughout this study was primarily sourced from Sigma-Aldrich Chemical Company (St. Louis, USA), except where specifically noted for alternative origins or suppliers. This study was conducted in accordance with the ethical guidelines of the Declaration of Helsinki. This study, performed on cosmetic products within the definition of article L. 5131-1 of the French Public Health Code, was conducted in accordance with Decree no. 2017–884 of 9 May 2017, modifying some regulatory requirements concerning studies involving human participants. All participants provided written informed consent and consent for photography before the start of the study. Written informed consent was obtained from the patients for the publication of this paper.

### 5.2. Callus Induction and Culture of FR

The FR flowers were soaked in 70% ethanol for 30 s, followed by washing with distilled water. The flowers were then shaken in 0.3% sodium hypochlorite (Waco) for 20 min and washed with distilled water. Next, they were cut into small pieces (petals) (0.5–1 cm) under aseptic conditions, followed by the culturing of the early stages of the plant cells in Murashige and Skoog (MS) medium (M0222; Duchefa, Haarlem, The Netherlands) with plant growth regulators, auxin and cytokinin, in the dark at 25 ± 2 °C. The first induced plant cells were propagated in the same Petri dish for 2–3 weeks. From the eighth week, the optimal combination ratio of dichlorophenoxyacetic acid (2,4-D) (D0911; Duchefa) was chosen based on the color, shape, and degree of differentiation of the plant cells. The selected plant cell line or callus was propagated in the optimal medium (pH 5.8 in a Petri dish; the composition of the medium is listed in Table S1). Thereafter, the calli were cultured in a bioreactor at the Plant Cell Research Institute of BIO-FD&C Co., Ltd. (Incheon, Republic of Korea). The harvested plant cells were subjected to extraction using distilled water at 40 °C for 4 h. For extract preparations, 2 g/L callus was used. After heat extraction, the solids were removed by filtration through a mesh. The FR callus induction process is briefly shown in Figure 1a.

### 5.3. Chemical Profiling through CPC and NMR Analysis

The methods for sample preparation are described in Section 1 of the Materials and Methods in the Supplementary Information. Briefly, an aliquot of each fraction from F01 to F08 from FCE obtained by CPC was dissolved in 600 µL of DMSO-d6 and analyzed by ^13^C NMR at 298 K on a Bruker Avance AVIII-600 spectrometer (Karlsruhe, Germany) equipped with a cryoprobe. After spectral processing, the absolute intensities of all the ^13^C NMR signals were automatically collected and binned across the spectra of the fraction series using a locally developed computer script. The resulting table was imported into PermutMatrix software (version 1.9.3, LIRMM, Montpellier, France) for hierarchical clustering analysis (HCA). The ^13^C NMR chemical shift clusters were visualized as dendrograms on a two-dimensional map. For metabolite identification, each ^13^C NMR chemical shift cluster obtained from HCA was manually submitted to the structure search engine of the database management software ACD/NMR Workbook Suite 2012 (ACD/Labs, Toronto, ON, Canada), which comprises the structures and predicted chemical shifts of low-molecular-weight natural products. In addition, 2D NMR experiments (HSQC, HMBC, and COSY) were also performed on all fractions to confirm or further elucidate the chemical structures proposed by the database at the end of the dereplication process.

### 5.4. Liquid Chromatography/Mass Spectrometry (LC/MS) Analyses

FCE was also analyzed by LC-MS to identify other minor metabolites that were not elucidated by NMR. The HPLC column used was a Uptisphere C-18 ODB 150 × 4.6 mm, 5 µm (Interchim), with a mobile phase consisting of MilliQ H_2_O + 0.1% formic acid for phase A and CH_3_CN + 0.1% formic acid for phase B. The flow rate was set at 0.7 mL/min, with a column temperature of 35 °C, a sample temperature of 20 °C, and an injection volume of 5 µL. The LC gradient ranged from 80% A at 0 min to 80% A at 25–30 min. The mass spectrometry parameters included ESI mode, scanning *m*/*z* 50–2000, with a scan rate of 1 s. Data processing was conducted using MassLynx 4.2 software.

### 5.5. Quantification of Nicotinamide by HPLC Coupled with LC/MS and Pyroglutamic Acid by LC/UV

Details on the process of nicotinamide sample preparation are provided and described in Section 2 of the Materials and Methods in the Supplementary Information. For nicotinamide quantification, the liquid chromatography parameters were employed using an Acclaim Polar Advantage II column (150 mm × 4.6 mm × 3 µm), operated at a column temperature of 40 °C, with an injection volume of 10 µL and a sample temperature of 15 °C. The mobile phase, described in Table S2, consisted of phase A (water with 20 mM ammonium formate salt and 0.1% formic acid) and B (methanol). Mass spectrometry parameters include an electrospray ionization source operating in positive mode with Selected Reaction Monitoring (SRM) acquisition mode, featuring a cone tension of 3500 Volt, sheath gas at 60, aux gas at 15, sweep gas at 2, and transfer tube and vaporization temperatures both set to 350 °C (Table S3).

Details on the process of pyroglutamic acid sample preparation are provided in Section 3 of the Materials and Methods in the Supplementary Information. For the quantification of pyroglutamic acid, an Acclaim Polar Advantage II column (150 mm × 4.6 mm × 3 µm) was maintained at a column temperature of 40 °C, with an injection volume of 10 µL, a sample temperature of 25 °C, and UV detection at 195 nm. The mobile phase, as detailed in Table S4, comprised phase A (water with 0.1% orthophosphoric acid) and B (acetonitrile).

### 5.6. Pro-Collagen I Synthesis

#### 5.6.1. Preparation of Cells and FCE Treatment

Primary normal human dermal fibroblasts (NHDFs) were plated in triplicate in a 6-well plate. The cells were cultured for 48 h in supplemented complete medium (DMEM medium; Gibco, Billings, MT, USA) at 37 °C with 5% CO_2_. Following the incubation period, cells designated for the prematurely “aged” condition were treated with hydroperoxide solution (H_2_O_2_) (Sigma-Aldrich, Saint Louis, MO, USA) for 2 h at 37 °C with 5% CO_2_. Subsequently, cells from both the “young” and “aged” conditions were washed twice with PBS (Gibco) and cultured for an additional 48 h in a basal medium supplemented with a positive reference mix containing TGF-β (T7039; Sigma-Aldrich) and ascorbic acid (A4034; Sigma-Aldrich), or with FCE at 0.3%. Untreated skin cells served as negative controls. Following a subsequent 48 h incubation period at 37 °C with 5% CO_2_, cell supernatants were collected and stored at −20 °C for pro-collagen I analysis.

#### 5.6.2. Type I Pro-Collagen Quantification Using ELISA

Type I pro-collagen was quantified using a Human Pro-Collagen I Alpha 1 ELISA Kit (ab210966; Abcam, Cambridge, UK). Briefly, the supernatants were diluted in the sample diluent. Then, the samples, along with the standard range, were incubated for 1 h in a 96-well plate pre-coated with type I procollagen antibodies in the presence of a cocktail antibody. After three washes with the wash buffer provided by the supplier, the TMB substrate solution was added to the wells and incubated for 10 min in the dark. Catalysis of this substrate by HRP results in a blue color that is proportional to the amount of type I pro-collagen bound in the initial step. Color development was halted by the addition of a stop solution, causing a change to a yellow color. Optical density was measured at 450 nm and 540 nm using a microplate reader (TECAN SPARK^®^ 10M). Finally, the data were analyzed using four-parameter logistic curve analysis.

### 5.7. Immunostaining of F-Actin on Fibroblasts in Collagen Lattices

#### 5.7.1. Preparation of Cells and FCE Treatment

The experiment was conducted using primary NHDFs in quadruplicate. Following amplification, NHDFs were harvested using trypsin solution and approximately 4 × 10^5^ cells/mL were combined with collagen. The cell–collagen mixture was then applied to each well in a 24-well plate. The plate was subsequently incubated at 37 °C with 5% CO_2_ for one hour to allow for collagen polymerization. After the collagen solidified, 1 mL of culture medium with or without 1% FCE was added. The incubation period lasted for two days, during which stress actin fibers developed. Matrices were released from the wells to induce collagen contraction. Following two days of contraction, the matrices were fixed for further analysis.

#### 5.7.2. Matrix Contraction Measurement

The matrix was detached from the wells and the collagen gel was inspected at different time intervals (2 and 4 h) using a camera (Huawei, China). The area of the matrices was quantified (in cm^2^) using ImageJ software (Java 1.6), and the results are presented as the mean ± standard error (SE).

#### 5.7.3. Immunostaining of F-Actin

The matrices were permeabilized with 0.1% Triton X-100 (T8787; Dutscher, Bernolsheim, France) in PBS for 5 min at room temperature and treated with an antibody targeting F-actin linked to a red fluorescent phalloidin conjugate (1/1000) (AB176757; Abcam, Cambridge, UK), according to the supplier’s recommendations. Additionally, the nuclei were labeled using DAPI (1/10,000) (Invitrogen, Carlsbad, CA, USA). Fluorescence imaging was performed using a confocal microscope (LSM780; ZEISS, Oberkochen, Germany). The average fluorescence intensity in each image was measured. The cell count in each image and the overall intensity were calculated to ascertain the average fluorescence intensity per cell.

### 5.8. Clinical Evaluation of FCE at 3%

#### 5.8.1. Panel Description

We conducted a clinical trial involving 43 female volunteers who were divided into two groups: group 1 was administered the placebo cream and consisted of 22 women aged approximately 46 years, whereas group 2 was administered the active cream and comprised 21 volunteers with an average age of approximately 47 years. All participants provided informed consent according to the guidelines outlined in the Declaration of Helsinki and the French Code de la Santé Publique Act on 20 December 1988. The cream formula contained 3% and was applied twice-daily for 28 days. Our assessment focused on evaluating the anti-aging properties of the active ingredient by analyzing skin roughness and collagen density after a 28-day application period.

#### 5.8.2. International Nomenclature of Cosmetic Ingredients (INCI) Formula

The creams used for testing in this study were as follows: aqua/water, cetyl alcohol, glyceryl stearate, PEG-75 stearate, Ceteth-20, Steareth-20, Isodecyl neopentanoate with or without FCE, phenoxyethanol, dimethicone, and fragrance.

#### 5.8.3. Analysis of Skin Roughness Using ColorFace^®^

The ColorFace^®^ system (Newtone Technologies, Lyon, France) is a 2D imaging setup designed for standardized multi-modality photos of the entire face. It features a 24-megapixel sensor and offers acquisition through various filters: ultraviolet, normal (filter-free), cross-polarized, parallel-polarized, standard photos at 45° angles, and standard photos at 60° angles. Using this system, digital facial photographs were taken on days D0 and D28, capturing images from the front, left, and right sides. Subsequently, skin roughness analysis was conducted by our partner, Spin Control in Tours, France, using ColorFace^®^ acquisitions and specialized imaging software.

#### 5.8.4. Collagen Index Measurement by Siascope^®^

The purpose of this experiment was to confirm the first results by performing a clinical evaluation of 43 female volunteers with irregular skin surfaces related to aging. Volunteers diligently applied the cream-based product for 28 days, following a regimen of twice-daily application in the morning and evening. The analysis of skin roughness, conducted using ColorFace^®^ analysis, allowed us to assess the impact of the product on the skin’s surface properties. Additionally, we employed SIAscope^®^ to measure the collagen index and evaluate the product’s effect on collagen production.

SIAscope employs a rapid, painless, and non-invasive technique for gauging hemoglobin, melanin, and collagen levels in the stratum corneum, skin, and dermis, reaching depths of up to 2 mm. This methodology capitalizes on the interaction between light and the skin. As light interacts with cells, it scatters or reflects the cellular absorption. This approach enables a comprehensive skin analysis using high-throughput spectrophotometric methods. Light simulations replicated diverse combinations of hemoglobin, melanin, collagen, and dermal melanin. The outcomes from each simulation were recorded and collectively examined to generate a siascan, a visual representation of the concentration of each chromophore per pixel. In this study, SIAscope^®^ measurements were taken at D0 and D28 along the jawline, specifically focusing on analyzing the “collagen” parameter.

### 5.9. Transcriptome Analysis

Two experimental groups were utilized for total RNA extraction: keratinocytes treated with DW (control) and keratinocytes treated with 1% FCE (experimental group). The total RNA was obtained from three different replicates of the cell cultures and then used to form cDNA libraries using the TruSeq Stranded mRNA LT Sample Prep Kit. The process, which was performed in technical triplicates, involved selecting poly A RNA, fragmenting RNA, performing reverse transcription with random hexamer priming, and sequencing with 100 nt paired-ends using Illumina NovaSeq 6000. Libraries were quantified by qPCR following the qPCR Quantification Protocol Guide, and their quality was assessed using an Agilent Technologies 2100 Bioanalyzer. Subsequently, raw reads were pre-processed to eliminate low-quality sequences and adapters before alignment to FR using HISAT v2.1.0 [61], which employs global and local indices constructed from the same BWT/graph FM index as Bowtie2. The FR reference genome and annotation data were obtained from the NCBI for Biotechnology Information database. StringTie v1.3.4d [62,63] was used to process the transcript assembly of known transcripts, allowing for the calculation of transcript and gene expression abundance as read counts or Fragments Per Kilobase of exon per million fragments mapped (FPKM) values per sample. These expression profiles facilitated further analyses, including the identification of DEGs, through statistical hypothesis testing in groups with distinct conditions.

### 5.10. Statistical Analysis

For both the in vitro and clinical data, data normality was first verified with Gaussian law using the Shapiro–Wilk test. Based on the results, we used unpaired or paired Student’s *t*-test parametric tests or Wilcoxon and Mann–Whitney nonparametric tests to compare the effect of FCE versus the untreated condition or placebo. For RNA sequencing, three biological and technical replications were applied and StringTie was used to gauge the relative gene abundance in the read counts. Subsequently, we conducted statistical analysis to identify DEGs by employing abundance estimates from each gene across the samples. Genes with zero or fewer reads were excluded. The refined information was subjected to log2 transformation and TMM Normalization. The statistical significance of differential expression was assessed using the edgeR exact Test [64] and fold change, wherein the null hypothesis assumed no differences between groups. To control for the False Discovery Rate (FDR), *p*-values were adjusted using the Benjamini–Hochberg algorithm. The set of DEGs underwent hierarchical clustering analysis using complete linkage and Euclidean distance for similarity measurement. Additionally, g:Profiler was employed for Gene Ontology (GO) analysis to gain further insights into the DEGs [65].

## Figures and Tables

**Table 1:** Mass and global composition of the CPC fractions. Maj = Major; Med = Medium; Min = Minor.

CPC- Fractions	Mass (mg)	Concentrated Extract (%)	Composition
01 Elution	4	0.2	Mixture of fatty acids and fatty alcohols (Maj); terephtalic acid (Min)
02 Elution	3	0.2	Hydroquinol–glucopyranoside (Maj); mixture of fatty acids and fatty alcohols (Med)
03 Elution	1	0.1	Mixture of fatty acids and fatty alcohols (Med);
04 Elution	2	0.1	Mixture of fatty acids and fatty alcohols (Med); nicotinamide (Min)
05 Elution	12	0.6	Methyl-pyroglutamate (Maj); Nicotinamide (Med)
06 Elution	45	2.4	Methyl-pyroglutamate (Maj); succinic acid (Maj); 4-(Glucopyranosyloxy)benzoic acid (Med); lactic acid (Min); malic acid (Min); pyroglutamic acid (Min); thymidine (Min); alanine (Min-)
07 Extrusion	1735	94.4	Sucrose (Maj++); glycerol (Maj); alanine (Med)
08 Extrusion	35	1.9	2′-Deoxyguanosine (Maj); 5-(1,2-dihydroxyethyl)-3-hydroxy-4-methoxyoxolan-2-one (Maj); adenosine (Med); sucrose (Med); uridine (Med); choline (Min); glycerol (Min); guanosine (Min); pyroglutamic acid (Min)

**Table 2:** LC/MS BPI chromatogram (ESI) of FCE.

LC Retention Time (min)	Observed *m*/*z*	Elemental Composition	Δppm	Annotation
2.09	173.1037 [M-H]^−^	C_6_H_13_N_4_O_2_	−1.2	Arginine
2.41	293.0981	C_10_H_17_N_2_O_8_	−1.4	*Not assigned*
2.83	194.0489	C_6_H_12_NO_4_S	1.0	*Not assigned*
2.94	191.0554 [M-H]^−^	C_7_H_11_O_6_	−1.0	5-(1,2-dihydroxyethyl)-3-hydroxy-4-methoxyoxolan-2-one *
3.11	341.1086 [M-H]^−^ 387.1136 [M+HCOO]^−^	C_12_H_21_O_11_ C_13_H_23_O_13_	0.6 −0.8	Sucrose *
3.52	229.0480	-	-	*Not assigned*
3.70	133.0137 [M-H]^−^	C_4_H_5_O_5_	0.0	Malic acid *
4.59	549.1663	-	-	*Not assigned*
4.64	191.0192 [M-H]^−^	C_6_H_7_O_7_	0.0	Citric acid
4.69	581.1831	-	-	*Not assigned*
4.71	128.0351 [M-H]^−^	C_5_H_6_NO_3_	2.3	Pyroglutamic acid *
4.74	243.0610 [M-H]^−^	C_9_H_11_N_2_O_6_	−2.9	Uridine *
4.87	282.0839 [M-H]^−^ 150.0416 fragment	C_10_H_12_N_5_O_5_ C_5_H_4_N_5_O	0.4 0.0	Guanosine *
4.93	266.0894 [M-H]^−^	C_10_H_12_N_5_O_4_	1.9	Adenosine *
5.00	287.0443	C_8_H_17_O_7_P_2_	2.4	*Not assigned*
5.38	299.0771 [M-H]^−^	C_13_H_15_O_8_	1.3	4-(glucopyranosyloxy)benzoic acid *
5.40	241.0824 [M-H]^−^	C_10_H_13_N_2_O_5_	−1.2	Thymidine *
7.55	439.1813	C_18_H_32_O_12_	1.6	*Not assigned*
14.20	329.2331	C_18_H_33_O_5_	0.6	C18 fatty acid derivative
23.44	567.3688	C_35_H_51_O_6_	0.4	*Not assigned*
24.80	481.3323	C_31_H_45_O_4_	1.0	*Not assigned*

* Compounds that were additionally identified by NMR.

## Data Availability

All data generated in this review are included in this paper. Further inquiries can be directed to the corresponding author.

## Supplementary Materials

The following supporting information can be downloaded at: https://www.mdpi.com/article/10.3390/ijms25158150/s1.
