# Supplementary material for: Analysis of Active Components and Transcriptome of Freesia refracta Callus Extract and Its Effects against Oxidative Stress and Wrinkles in Skin
Source: Int J Mol Sci. 2024 Jul 26;25(15):8150. doi: 10.3390/ijms25158150 (PMC11311438; doi:10.3390/ijms25158150)
Supplement: Supplementary file 1 [file ijms-25-08150-s001.zip › ijms-3076972-supplementary.pdf]

Supplementary data

Analysis of active components and transcriptome of *Freesia retracta* callus extract and its effects against oxidative stress and wrinkles in skin

Euihyun Kim[1], Morgane De Tollanaere[2], Benedicte Sennelier[3], Carole Lambert[4], Anais Durduret[2], Soo Yun Kim[1], Hyo Hyun Seo[1], Jung Hun Lee[1], Amandine Scandolera[2], Romain Reynaud[4], Sang Hyun Moh[1Ŧ]

[1] Plant cell Research Institute of BIO-FD&C Co. Ltd., Incheon 21990, Korea
[2] Givaudan France SAS, Route de Bazancourt 51110 Pomacle, France
[3] Givaudan France Naturals, 250 rue Pierre Bayle, BP 81218, 84911 Avignon, France
[4] Givaudan France SAS, Bâtiment Canal Biotech 1, 3, Rue des Satellites, 31400 Toulouse, France

[Ŧ]**Corresponding author:** Sang Hyun Moh, biofdnc@gmail.com (S.H.M), TEL: +82-32-816-2027

Materials and methods

1.Sample Preparation for NMR and LC/MS Analysis

1.1. Chemical profiling

Chemical profiling was performed by dereplication through centrifugal partition chromatography coupled with NMR ^{13}C (CPC-NMR).

1.2 Sample preparation

The sample (batch number: S.BFR20220316022) was characterized using a liquid containing 0.76% *FR* callus cell extract, 29% propane-1.3-diol, and 70.24% water. Approximately 85 g of the starting extract was dried at 60°C (0.1 mbar) for 48 h using a Genevac™ apparatus to reduce the amount of 1,3-propanediol. An extract of 2.34 g was obtained.

1.3 Extract fractionation by CPC

The concentrated extract of 2.34 mg was injected into the FCPC200® instrument (Rousselet Robatel Kromaton) equipped with a 260 mL (800 cells) column, utilizing two-phase solvent systems 1 and 2 consisting of Methyl-ter-butyl-ether/n-butanol/water (4/1/5, v/v/v) and (1/4/5, v/v/v), respectively, with the stationary phase being the lower phase of the two-phase solvent system 1 in ascending mode. The column rotation speed was set to 1300 rpm at a flow rate of 12 mL/min, according to the mobile phase gradient (Table S2). The column was filled with the lower phase of System 1 and equilibrated with the upper phase of System 1 at 12 mL/min and 1500 rpm. The sample was dissolved in 10 mL of the lower phase and 7 mL of the upper phase of System 1 and injected into the CPC column using a 20-mL loop. Fractions of 20 mL were collected during the entire experiment (elution and extrusion) and combined according to their thin-layer chromatography (TLC) profiles. TLC was performed on pre-coated silica gel 60 F254 Merck plates with an ethyl acetate/acetic acid/formic acid migration acid (10/1/1, v/v/v), visualized under UV light at 254 nm and 366 nm, and revealed by spraying the dried plates with 50% H_2SO_4 and vanillin, followed by heating. As a result, 10 fractions were obtained. Some elution fractions were recovered in minimal quantities; therefore, F04+F05 and F06+F07 were combined, resulting in a final series of n eight fractions (Figure 2a-c). Eight final fractions were used for identification (NMR and LC/MS analyses).

2. Quantification of Nicotinamide by High-Performance Liquid Chromatography Coupled with MS/MS Detection (HPLC-MS-MS)

2.1 Sample Preparation

The sample (batch number: 2309-E0151562) was a water solution containing 1% dry matter of FCE. A 100 g/L solution was prepared by weighing an exact mass of approximately 100 mg of the sample in a 1-mL vial. The solution was supplemented with a mixture of water/methanol (50/50, v/v) and 0.1% formic acid. The solution was prepared in duplicate. A solution spiked at 10 µg/L was prepared similarly but by adding 100 µL of the standard niacinamide working solution at 100 µg/L.

2.2 Standard and Calibration Curve

The analytical standard used was niacinamide (syn: nicotinamide; n° CAS: 98-92-0). A stock solution at 1 g/L calibration was prepared by weighing approximately 10 mg of the analytical standard in a 10 mL vial. This solution was supplemented with water/methanol (50/50, v/v) and 0.1% formic acid. Working solutions at 10 mg/L and 100 µg/L are prepared from the stock solution. The calibration range extends from 2 µg/L to 100 µg/L. The samples were injected into a Thermo Vanquish apparatus equipped with a Thermo Altis Plus mass spectrometer.

3. Quantification of Pyroglutamic Acid by High-Performance Liquid Chromatography Coupled with UV

Detection (HPLC-UV)

3.1 Sample Preparation

The sample (batch number: 2309-E0151562) was a water solution containing 1% dry matter of FCE. A 100 g/L solution was prepared by weighing an exact mass of approximately 100 mg of the sample in a 1-mL vial. The medium was then supplemented with water. The solution was prepared in duplicate. A solution spiked at 50 mg/L was prepared in the same way with the exception of the addition of 100 μL of the standard working solution at 500 mg/L.

3.2 Standard and Calibration Curve

Analytical grade pyroglutamic acid was used (CAS: 98-79-3). Stock solutions in the 1 g/L calibration and quality control ranges were prepared by weighing approximately 10 mg of the analytical standard in a 10-mL vial. This was supplemented by EUP. A working solution at 500 mg/L was prepared from the stock solution. The calibration range extended from 5 mg/L to 200 mg/L. The samples were injected into a Thermo Vanquish apparatus equipped with UV detector.

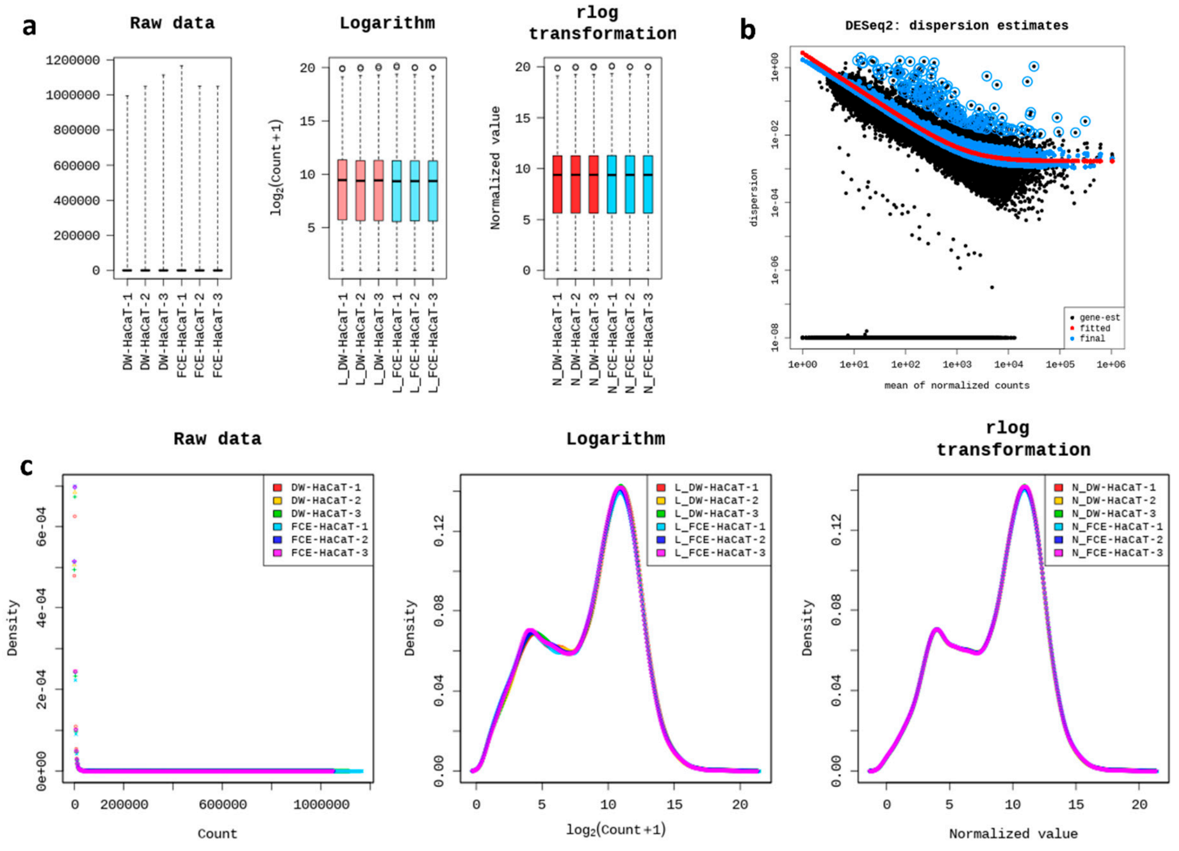

Figure S1. Trimming and normalization of raw data. (a and c) The data were logarithmized and normalized, then (b) the mean of normalized counts was visualized by dispersion. DW: Distilled water; FCE, Freesia callus extract.

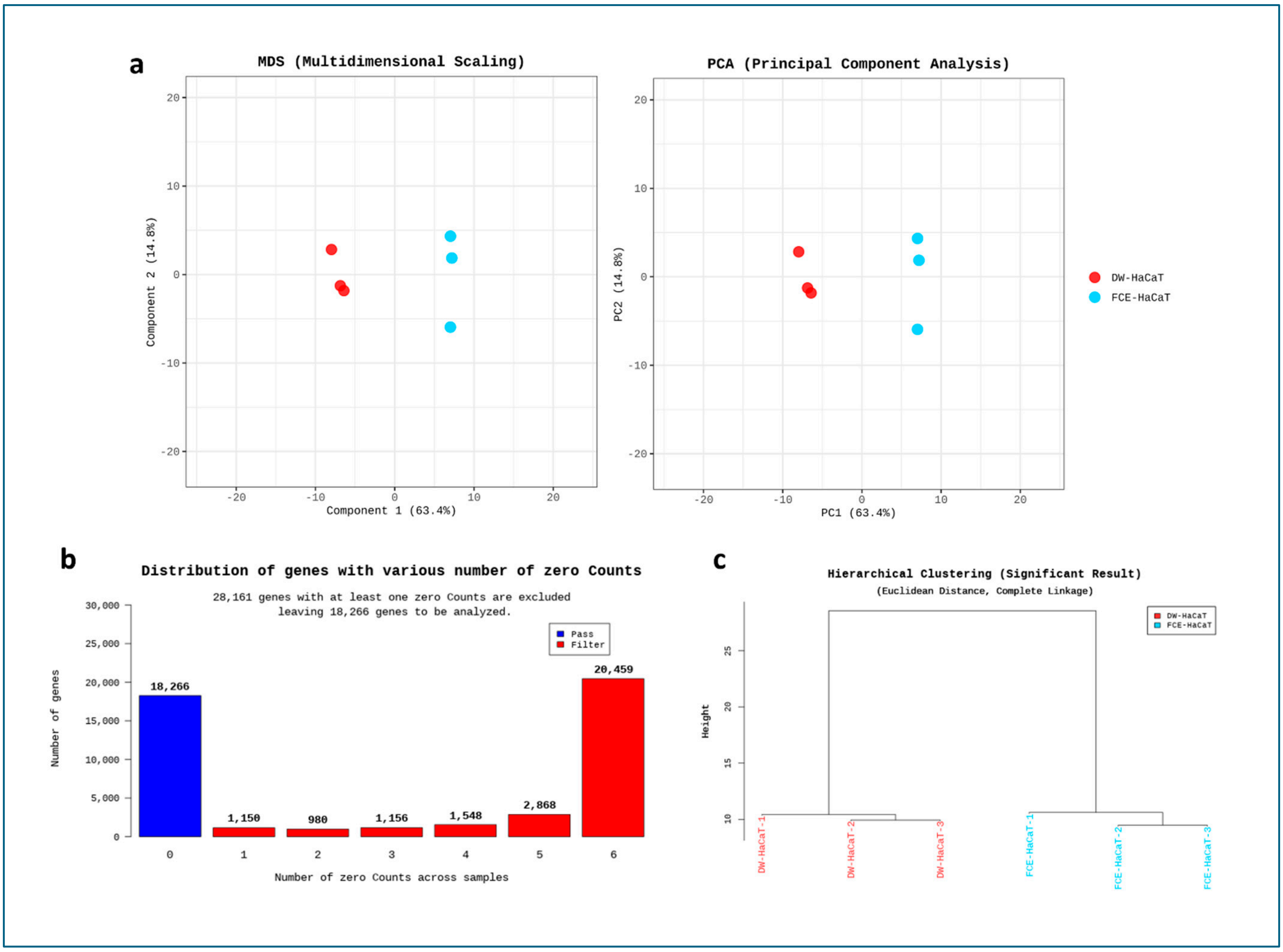

Figure S2. Three replicates of each experimental group were primarily compared using (a) multidimensional scaling and principal component analysis. (b) In cases where genes had zero counts, a total of 28,161 genes with at least one zero count were excluded, and 18,266 genes were analyzed. (c) Each experimental group was categorized by hierarchical clustering. DW: Distilled water; FCE: Freesia callus extract.

Table S1. Composition of media for FR callus culture.

Components	mg/L
NH_4NO_3	1,650.0
KNO_3	1,900.0
$MgSO_4·7H_2O$	370.0
$MnSO_4·4H_2O$	22.3
$ZnSO_4·7H_2O$	8.6
$CuSO_4·5H_2O$	0.025
KH_2PO_4	170.0
KI	0.83
$CoCl_2·6H_2O$	0.025
$CaCl_2·2H_2O$	440.0
H_3BO_3	6.2
$Na_2MoO_4·2H_2O$	0.25
$FeSO_4·7H_2O$	27.85
$Na_2·EDTA$	37.25
Thiamine HCl	0.4
Myo-inositol	100.0
α-naphthalaneacetic acid	2.0
6-benzyladenine	1.0
Sucrose	30,000.0
Gelrite	2,300.0
pH adjusted to 5.8<br>Sterilized at 121℃ for 15 min	

Table S2. Mobile phase conditions for nicotinamide sample preparation.

Time (min)	Flow rate (mL/min)	% A*	% B**
0.0	0.8	99%	1%
0.5	0.8	99%	1%
3	0.8	5%	95%
6.5	0.8	99%	1%
6.6	0.8	99%	1%
8.0	0.8	99%	1%

* Water with 0.1% orthophosphoric acid

** Acetonitrile.

Table S3. Experimental conditions for mass spectrometry and selected reaction monitoring (SRM).

**Mass spectrometry**	
Ionisation source	Electrospray
Ionisation mode	positive
Acquisition mode	SRM
Cone tension	3500 Volt
Sheath gas	60
Aux gas	15
Sweep gas	2
Transfer tube temperature	350°C
Vaporization temperature	350°C
**SRM**	
Retention time (min)	4.6
Parent Ion (m/z)	122.9
Quantification Ion (m/z)	77.9
Confirmation Ion (m/z)	52.9

Table S4. Mobile phase conditions for pyroglutamic acid sample preparation.

Time (min)	Flow rate (mL/min)	% A*	% B**
0.0	0.7	100%	10%
8.0	0.7	0%	100%
11.0	0.7	0%	100%
11.1	0.7	100%	0%
16.0	0.7	100%	0%

* Water with 0.1% orthophosphoric acid

** Acetonitrile.
